# Methods of Feces Disposal and Fly Prevention in a Division

**Published:** 1918-10

**Authors:** J. W. Gressinger


**Feces Disposal and Fly Prevention in a Division.** Colonel J. W. GRESSINGER, M. C. The speaker said in part :

This subject has been so thoroughly discussed by those who have preceded me on the program that I feel that there is very little left for me to say.

There are several points, however, which, though mentioned, I feel have not been sufficiently emphasized. It seems worth while to draw special attention to them. The latrine boxes, incinerators, buckets, grease traps, and other appliances so clearly and fully described by Major Joscelyn of the British Army, are all admirable and splendidly adapted to the purpose intended. I can add nothing to them. However, in the sector occupied by American troops, transportation is such a serious problem and there is such a shortage of lumber that these appliances cannot be used. We from the States know that the Havard box properly cared for and burned out daily answers the problem perfectly. But we can get no oil and not sufficient lumber so that we must make the best of circumstances and utilize some other means of feces disposal.

In sector warfare, we have found that a flat topped lid made by nailing boards over two cross pieces and just big enough to properly cover the latrine pit works very well. Every second or third board, depending upon their width, is not nailed down but is furnished with a handle accurately fitted into place, and makes the lid for the corresponding opening. There are several distinct advantages of this type of cover over the Havard box :

*a*) It requires only one fourth the lumber.

*b*) It has only one surface to be made fly proof instead of five.

*c*) There is no contact of the men with the seat thereby lessening the chance of spread of vermin and possibly also of venereal disease. The men must assume a squatting position but this is natural and not a disadvantage.

Latrine buckets can also be used with good results if properly cleaned and cared for. The straddle trench should have no place in trench warfare, as enough lumber can usually be secured to make the flat top cover mentioned above. Failing this, latrine buckets can be used. Incineration has been out of the question. Fuel is scarce and oil so far has been unobtainable in any quantity that would be of practical value. Also the smoke by day and light by night cause considerable danger from enemy shell fire.

So far, in the American advanced areas, we have been unable to use ideal methods but have been compelled to improvise and do the best possible with what we could procure.

The main point, however, that I want to emphasize strongly is that it is not a question of type. It is a question of personal supervision. It doesn't matter a particle what method is employed provided ordinary judgment is used in the selection of the particular method. Anyone of the methods mentioned will be perfectly satisfactory if proper supervision is given it; any one of them will be an utter failure if not properly supervised. We have seen untrained, undisciplined troops, with untrained officers occupy a modern barrack with a modern toilet, tiled floor and flush closets, and have seen it in a week transformed into a filthy, nauseating mess. Newspapers being thrown into the flush bowls, clogging the pipes, the chain when pulled gave an overflow with easily imagined results, toilet-paper and newspaper all over the floor; in general as unsanitary a place as one could well imagine.

On the other hand, we have seen a company go into the field with nothing but shovels for sanitary appliances, and conduct a camp that was entirely sanitary; feces disposed of in straddle trenches, promptly covered, attracting no flies and giving rise to no offense.

We have seen on the Mexican border a well disciplined organization have latrines with fly tight boxes, protected by a screened shelter, that were a delight from a sanitary viewpoint. Burned out daily, kept immaculately clean and perfectly fly proof, there was no odor, no flies, and one could have stayed in the enclosure for an hour scarcely realizing that a latrine was in the vicinity.

Within two miles of the same spot, we have seen the same facilities: Havard box, screened shelter, oil and straw for burning; but in a week this latrine was unspeakably dirty, paper scattered everywhere, urine trough foul, box not fly-proof, and the contents of the latrine pit alive with maggots, with flies thick in the enclosure.

Here were identically the same facilities with exactly opposite results.

I repeat, as strongly as I am able to express it, that it does not matter a particle what system one adopts. Proper supervision is the whole solution.

To secure results there must be —

A. Education.

We, as medical officers, understand the dangers of disease transmission by flies, but it is astonishing how many line officers do not realize its importance. The enlisted men as a mass do not have the proper realization of the problem and there are also some medical officers who need instruction in the matter.

Education is then a most important function so that officers and men may have a proper realization of the dangers connected with this pest.

B. Organization — Discipline.

Without organization and discipline, we get nowhere. With them, we can accomplish anything. Some organizations have officers at their head who are alive to this subject, interested in the welfare of their men and always anxious to obtain good advice. With these there will be no trouble, but their number is far too small. The majority require constant supervision in order to prevent serious outbreaks of preventable diseases.

C. Unceasing Vigilance.

This requires constant inspections no matter how irksome they may become. Make the inspections at unexpected times.

Place the responsibility for each particular thing on some particular individual and under no circumstances accept excuses. If you do, there will always be a plausible one presented.

Be liberal with praise where good work is being done. We are all human and it is astonishing how hard men will work if they feel that their efforts are really appreciated. They soon come to take such a pride in the organization that it well repays all effort expended. On the other hand, follow each sanitary offense with appropriate punishment as inexorably as night follows day.

There are too many organizations where, when the sanitary inspector reports a dirty latrine to the organization commander, the latter calls the lieutenant and transmits the report telling him to have it corrected. The lieutenant calls a sergeant and passes on the instructions, the sergeant repeats the same to a corporal, the corporals picks out the nearest private and tells him to get a broom and clean the place. The private has nobody to fall back on, so he does the work. Perhaps the captain inspects the latrine next day and finds it clean. He throws out his chest, thinks he has discipline and promptly forgets the matter. At the next visit of the sanitary inspector, conditions are just as bad as before and when he reports to the company commander the latter cannot understand how that can be possible. The point is, there must be *unceasing vigilance*, constant inspections and untiring personal supervision of the company commander and the surgeon. With this results will be perfect no matter what method is used. With anything less, any method will be a failure. This cannot be too strongly emphazised.

The disposal of garbage presents essentially the same features. It may be disposed of by incineration, burying, or by giving or selling to farmers. It is not necessary to go into details as to these methods, as they have been fully covered and I can presentn othing new. Each method is satisfactory if properly supervised. Each is insanitary if neglected.

Open Warfare:

In the recent second battle of the Marne, the writer, who had the honor of serving throughout that struggle, was impressed with several very striking sanitary problems.

1. The Germans in their retreat left things in foul condition. In barns and similar buildings, they had defecated promiscuously in rooms on the floor. I have been told by medical officers that they did the same in rooms of chateaux and that they even defecated in drawers. They also used the surface of the ground and left open latrine pits with no attempt to cover the contents. The result was that flies had bred in great numbers from causes over which we had no control.

2. Our own men, after undergoing the tremendous mental and physical strain of 4 to 6 days fighting, let down very markedly in sanitary matters. As they were brought back in reserve and camped in shelter tents in the woods, these men were utterly exhausted mentally and physically. All they wanted was rest, food and drink, and the simplest sanitary precautions were forgotten. Men defecated on the surface of the ground or, if pits were dug, the contents were not covered. The result was more flies.

There was of course marked disorganization in some outfits through loss of officers. One battalion, for instance, lost every officer; naturally that battalion deteriorated rapidly in a sanitary way from lack of supervision. There was also a too prevalent attitude among medical officers that "Well, we will be here for only a day or so" and no precautions were taken. They were kept longer than at first anticipated and soon had a most unsanitary camp.

Medical officers should be impressed with the fact that, no matter how temporary they think a camp will be, they must take every precaution to prevent fly breeding or any other unsanitary development. Always go on the supposition that your camp is going to be more or less permanent and no sanitary faults from this cause can then arise.

3. The most striking thing, however, was the number of dead bodies of men and animals and the length of time they remained on the field. They were scattered everywhere, and as they lay for days unburied the stench was terrific. It is believed many of these bodies were unburied for at least 10 days. Worse than the odor, however, was the fly breeding that took place in these bodies. They soon became black, swollen, decomposing masses of organic matter, simply alive with maggots. They were the principal cause of a plague of flies that it was necessary to see in order to properly appreciate.

There was direct and easy access from these bodies and open latrine pits to the food of officers and men, and the flies were not slow in taking full advantage of the fact. The result was just what would be expected — a widespread outbreak of diarrhea which affected nearly everybody. Fortunately, cases were mild in character and there was no mortality. Men were not sick usually more than 2 or 3 days but there was considerable tendency to recurrence.

Investigation by bacteriologists showed the presence of Shiga and Flexner bacilli, paratyphoid and certain aberrant types.

We were fortunate in getting away with a mild infection with no mortality, but there is no assurance that under similar circumstances in the future a true virulent Shiga type dysentery might not be implanted upon this highly favorable soil and a serious epidemic supervene with high mortality and tremendous ineffectiveness.

Were it not for typhoid and paratyphoid inoculation, under conditions such as existed during this recent campaign, there is no question but that these diseases would be rampant. But this inoculation presents no barrier to dysentery and vigorous steps must be taken to prevent such outbreaks in any future operations. It will take vigorous systematic methods to prevent disaster in the extensive open operations that we are apt to have next year during the warm season. The greatest menace lies in the delay in burial of dead men and animals. A systematic method must be instituted at once to overcome this.

As a solution, the writer proposes that each corps be furnished with a labor battalion — to begin with, say 5000 men. These men properly officered will follow up divisions as closely as possible and bury dead men and animals. They will work in conjunction with the Graves Registration Service in order to assure proper identification. In this way, the battle field can be kept clean, bodies buried promptly and fly breeding from this source entirely eliminated.

They should be corps troops, the division being left free for fighting purposes. The Pioneer Infantry of a corps could well be utilized for this purpose.

Sanitary inspectors, corps and division, will do well to carefully supervise this work which will be a most important function. Combatant troops should not for several reasons be called upon to bury their dead.

*a*) Combatant troops cannot be taken from the line during combat for this purpose. Prompt burial of the dead is absolutely essential.

*b*) Combatant troops cannot be called upon after combat as they are mentally and physically exhausted,

*c*) The effect of this kind of work on the morale of troops is the worst imaginable. Nothing worse can be imagined than to have combatant troops returning from a battle compelled to bury their comrades, especially if days have elapsed during warm weather and the bodies are in the condition noted above. Each man so engaged will have a mental picture of himself in exactly the same condition every subsequent time that he goes into action.

On the other hand, if troops coming back from combat see no evidence of the recent engagement, they will subconsciously conclude that it wasn't so bad after all. All wounded removed promptly and all dead decently and promptly buried will have a wonderful effect in maintaining the morale of troops. The men who are risking their lives in this tremendous conflict are entitled to nothing less.

