# Correction

**Published:** 1918-12

**Authors:** 


### CORRECTION

*In the issue of " War Medicine " for October 1918, p. 338, line 30, the strength of the labor battalion as proposed should be 500 instead of 5,000 men.*

